# Effectiveness of a multidisciplinary collaborative management model in the control of hospital-acquired infections caused by multidrug-resistant organisms

**DOI:** 10.1097/MD.0000000000045345

**Published:** 2025-11-14

**Authors:** Lan Ding, Fubo Gao

**Affiliations:** a Department of Infection Control, Dongcheng District First People’s Hospital of Beijing, Beijing, China.

**Keywords:** antimicrobial stewardship, hospital-acquired infections, infection control, multidisciplinary management, multidrug-resistant organisms

## Abstract

Hospital-acquired infections caused by multidrug-resistant organisms (MDROs) increase patient morbidity and healthcare costs. This study assessed a multidisciplinary collaborative management model to improve infection control and reduce MDRO rates. We conducted a retrospective cohort study at a tertiary hospital in Beijing from January 2021 to December 2024. Adult inpatients (≥18 years, length of stay ≥ 48 h) with complete clinical and microbiological data were included. Patients hospitalized before implementation of the multidisciplinary collaborative management model (January 2021–December 2022) comprised the control group, while those admitted after its launch (January 2023–December 2024) formed the intervention group. A multidisciplinary team – including infection control specialists, pharmacists, microbiologists, nursing staff, and ICU leadership – conducted twice-weekly ward rounds and supervised infection prevention practices. Compliance indicators, MDRO detection rates, and infection incidence were compared between groups. Compliance with infection control measures improved significantly after implementation (e.g., training 78.3% → 97.5%, environmental cleaning 83.3% → 95.7%, PPE use 86.4% → 93.8%; all *P* < .001). The MDRO detection rate decreased from 60.1 to 52.5% (χ^2^ = 43.5, *P* < .001). Overall MDRO infection incidence declined from 0.05 to 0.02% (χ^2^ = 9.18, *P* = .003), with marked reductions in ICU and emergency department intensive care units. Implementation of a multidisciplinary collaborative management model significantly enhanced compliance with infection control measures and reduced MDRO prevalence and infection incidence, particularly in critical care units. This approach provides a practical strategy for strengthening hospital infection control programs.

## 1. Introduction

Hospital-acquired infections (HAIs) represent a significant burden on healthcare systems worldwide, contributing to increased morbidity, mortality, and economic costs. Among these, infections caused by multidrug-resistant organisms (MDROs) pose particular challenges due to limited therapeutic options and their potential for rapid dissemination within hospital environments. Common MDROs include methicillin-resistant staphylococcus aureus (MRSA), vancomycin-resistant enterococci, and extended-spectrum β-lactamase-producing gram-negative bacilli.^[1,2]^ The prevalence of MDRO-associated HAIs has risen steadily over the past decade, with reported rates ranging from 5 to 20% of all nosocomial infections depending on geographic region and institution type. This upward trend underscores the urgency of implementing effective infection prevention and control strategies tailored to resistant pathogens.

Traditional approaches to HAI control have largely relied on single-discipline interventions such as antimicrobial stewardship programs or environmental disinfection protocols.^[3,4]^ Although these measures can reduce overall infection rates, their effectiveness against MDROs is often limited by the multifactorial nature of resistance transmission. Overuse and misuse of broad-spectrum antibiotics drive resistance, lapses in hand hygiene and contact precautions facilitate cross-transmission, and structural barriers – including inadequate staffing, limited microbiology capacity, and fragmented communication – hinder timely identification and isolation of carriers.^[5,6]^ Recent studies have shown that single interventions yield only modest improvements: for example, a stewardship-only program reduced carbapenem-resistant Klebsiella pneumoniae by 12%, while adding coordinated infection control strategies achieved reductions exceeding 30%.^[7,8]^ These findings highlight the need for more integrated, multidisciplinary approaches.

To address these challenges, multidisciplinary collaborative management models have been proposed as a comprehensive framework that leverages expertise from infection control specialists, microbiologists, pharmacists, nursing leadership, and environmental services personnel. Core elements include real-time pathogen surveillance, standardized laboratory protocols, multidisciplinary case reviews, and antimicrobial regimen adjustments guided by local antibiogram data. Educational initiatives reinforce adherence to best practices such as hand hygiene, use of personal protective equipment (PPE), and environmental cleaning.^[9,10]^ Beyond frontline clinical care, administrative support, automated alert systems, and quality improvement metrics strengthen the sustainability of such models. However, despite encouraging reports, evidence from large-scale, structured hospital-wide evaluations remains limited.

This study was designed to test the hypothesis that implementation of a multidisciplinary collaborative management model can significantly improve adherence to infection control measures and reduce both the detection rate and infection incidence of MDROs. By addressing existing gaps in evidence, our findings aim to provide practical guidance for hospitals seeking to adopt integrated strategies for MDRO prevention and control.

## 2. Methods

### 2.1. Study design

A retrospective cohort study was conducted at our institution from January 2021 through December 2024. The Multidisciplinary Collaborative Management Model was launched on January 1, 2023; therefore, patients hospitalized between January 2021 and December 2022 served as the control cohort, while those admitted between January 2023 and December 2024 comprised the intervention cohort. Baseline characteristics of the 2 cohorts, including age, sex, and major comorbidities, were compared to assess group comparability, and no statistically significant differences were observed. The study protocol was developed and executed in strict accordance with the Declaration of Helsinki and received formal approval from the Dongcheng District First People’s Hospital of Beijing Medical Ethics Committee.

Inclusion criteria: age ≥ 18 years; hospitalization duration ≥ 48 hours to ensure adequate observation time for HAIs; availability of complete clinical, microbiological, and infection control records; and patients who underwent routine MDRO surveillance and were eligible for multidisciplinary ward rounds.

Exclusion criteria: patients < 18 years old; length of stay < 48 hours; readmissions of the same patient during the study period (only the first hospitalization was included to avoid duplication); patients transferred from other hospitals with documented MDRO infection prior to admission; and cases with incomplete clinical or microbiological data.

## 3. Multidisciplinary collaborative infection management model

A dedicated multidisciplinary team was convened to coordinate prevention and control of MDRO-related HAIs. Core membership comprised infection control specialists, representatives from the medical affairs and nursing departments, clinical pharmacists, microbiologists from the laboratory, information technology staff, environmental services managers (including the contracted cleaning service), and ICU leadership. Under the direction of the infection control department, this team develops and implements a twice-weekly MDRO ward-round schedule, with clearly defined roles and responsibilities assigned on each rounding day.

Prior to each inspection, team members review the clinical status of all inpatients with MDRO infections. Key parameters include vital signs (e.g., temperature, level of consciousness, presence of pressure ulcers), microbiological and diagnostic results, antimicrobial regimens (including indications and timing of pathogen sampling), catheter-associated device use, and prior MDRO detection data. For antimicrobial stewardship, the team assessed appropriateness of prescriptions based on pathogen culture and susceptibility results, monitored defined daily doses per 100 bed-days, and calculated the proportion of antibiotic adjustments guided by antibiogram data.

During ward rounds, the team evaluates adherence to MDRO prevention measures among high-risk populations – such as critically ill patients, those with indwelling devices, and individuals with prolonged hospitalization. Rounds begin with the early shift handover. They then include bedside verification of isolation precautions, review of physician orders and nursing documentation, and direct observation of environmental cleaning. Objective assessments employ surface sampling and fluorescent marker tests to quantify disinfection efficacy on linens and high-touch surfaces.

In parallel, structured staff training sessions were conducted monthly, covering topics such as hand hygiene, PPE usage, antimicrobial prescription standards, and environmental cleaning protocols. Training coverage rates were monitored (≥90% participation per department), and post-training assessments were required to ensure knowledge retention.

Findings from ward rounds and training were documented in a standardized “Infection Control Rounding Record,” which captures identified deficiencies, antimicrobial stewardship indicators, microbiological monitoring results, fluorescence audit outcomes, corrective actions, and on-site training interventions. Issues are managed through a PDCA (Plan–Do–Check–Act) cycle, with the infection control department responsible for tracking performance metrics and sustaining iterative improvement.

## 4. Data collection and outcome measures

A comprehensive set of process and clinical indicators was systematically collected to evaluate the impact of the multidisciplinary collaborative management model. Process measures included: isolation signage accuracy and visibility; use of patient-dedicated medical devices and adherence to device-specific decontamination protocols; thoroughness of environmental cleaning and terminal disinfection, as verified by fluorescence marker audits and adenosine triphosphate bioluminescence testing; frequency and quality of department-level training and completeness of early shift handover documentation; timeliness of MDRO exposure notifications during interdepartmental transfers and preoperative briefings; initiation and discontinuation of isolation orders in electronic medical records; compliance with recommended PPE use; and proper segregation, transport, and disposal of infectious waste and contaminated linens. Hand hygiene adherence at the bedside of MDRO-positive patients was assessed through direct observation using the World Health Organization “Five Moments” framework. Clinical outcome measures comprised: rates of MDRO recovery from surveillance specimens; and incidence density of MDRO-associated HAIs.

## 5. Statistical analysis

All statistical analyses were performed using IBM SPSS Statistics version 28.0 (IBM Corp., Armonk). Categorical data are reported as frequencies and percentages and were evaluated using the chi-square (χ^2^) test or Fisher exact test as appropriate. All tests were two-tailed, and a *P*-value of <.05 was considered indicative of statistical significance. To adjust for potential confounding factors (e.g., age, sex, length of stay, and ICU admission), multivariable logistic regression models were conducted for the main outcomes (MDRO detection and infection incidence).

To address missing data, we performed a complete-case analysis; cases with incomplete clinical or microbiological records were excluded according to the predefined exclusion criteria. Sensitivity analyses confirmed that the exclusion of these cases did not materially affect the results.

## 6. Results

### 6.1. Compliance with infection control measures

Following implementation of the multidisciplinary collaborative management model, overall adherence to prescribed infection control protocols improved markedly across all evaluated domains (Table 1). The most notable gains were observed in department-level training and early handover procedures (78.3% → 97.5%, *P* < .001), environmental cleaning and disinfection (83.3% → 95.7%, *P* < .001), and PPE use (86.4% → 93.8%, *P* < .001). Similar upward trends were also recorded in isolation management, equipment disinfection, transfer/surgical notifications, and medical waste disposal, as detailed in Table 1. These findings indicate that the integrated management framework significantly enhanced adherence to critical infection prevention practices.

**Table 1:** Compliance with infection control measures before and after implementation of the multidisciplinary collaborative management model.

Infection control measure	Pre-implementation (n = 516)	Post-implementation (n = 531)	χ^2^	*P*-value
Isolation signage	492 (95.3%)	525 (98.9%)	11.661	.001
Dedicated equipment & disinfection	477 (92.4%)	518 (97.6%)	14.485	.001
Environmental cleaning & disinfection	430 (83.3%)	508 (95.7%)	42.695	<.001
Department training & early handover	404 (78.3%)	518 (97.5%)	92.356	<.001
Transfer/surgery notification	478 (92.6%)	526 (99.1%)	27.415	<.001
Isolation order initiation/discontinuation	490 (95.0%)	522 (98.3%)	9.056	.003
PPE use	446 (86.4%)	498 (93.8%)	15.943	<.001
Medical waste & linen disposal	461 (89.3%)	527 (99.2%)	48.296	<.001

χ^2^ = chi-square test, n = number of patients, % = percentage, PPE = personal protective equipment.

## 7. MDRO detection rates before and after implementation

The overall detection rate of multidrug‐resistant organisms among clinical isolates decreased significantly following implementation of the multidisciplinary collaborative management model, from 60.1% (2267/3770) to 52.5% (1866/3555) (χ^2^ = 43.5, *P* < .001). Reductions were observed across all key MDRO species: carbapenem-resistant enterobacterales (CRE) declined from 38.9 to 34.7%, carbapenem-resistant acinetobacter baumannii from 13.8 to 11.8%, carbapenem-resistant pseudomonas aeruginosa from 3.8 to 3.0%, and MRSA from 3.6 to 2.9%. These downward trends suggest that the coordinated interventions – encompassing enhanced surveillance, targeted antimicrobial stewardship, and strengthened infection control practices – effectively curtailed the burden of MDRO colonization and infection within the hospital environment (Table 2).

**Table 2:** Comparison of MDRO detection rates in hospitalized patients before and after implementation of the multidisciplinary collaborative management model.

MDROs	Pre-implementation (surveyed isolates: 3770)	Detection rate (%)	Post-implementation (surveyed isolates: 3555)	Detection rate (%)	χ^2^	*P*-value
CRE	1465	38.9	1235	34.7	–	–
MRSA	135	3.6	104	2.9	–	–
CR-AB	522	13.8	419	11.8	–	–
CR-PA	145	3.8	108	3.0	–	–
Total	2267	60.1	1866	52.5	43.5	<.001

χ^2^ = chi-square test, CR-AB = carbapenem-resistant acinetobacter baumannii, CRE = carbapenem-resistant Enterobacterales, CR-PA = carbapenem-resistant pseudomonas aeruginosa, MRSA = methicillin-resistant Staphylococcus aureus.

## 8. Incidence of MDRO infections in ICU, ED-ICU, and general wards

Overall, the incidence of MDRO infections across the 3 monitored hospital units declined significantly following implementation of the multidisciplinary collaborative management model, decreasing from 0.05% (37/81,211) in the pre-implementation period to 0.02% (17/88,226) post-implementation (χ^2^ = 9.18, *P* = .003). When analyzed by unit, the intensive care unit (ICU) experienced a marked reduction in MDRO infection incidence – from 1.48% (16/1080) pre-implementation to 0.42% (5/1198) after implementation (χ^2^ = 7.04, *P* = .008). Similarly, the emergency department intensive care unit (ED-ICU) demonstrated a significant decrease, with incidence falling from 3.27% (5/153) to 0.27% (1/377) (χ^2^ = 8.77, *P* = .003). In contrast, the general wards maintained a low baseline incidence, with a nonsignificant change from 0.02% (16/79,978) to 0.01% (11/86,651) (χ^2^ = 1.37, *P* = .241) (Table 3). These findings indicate that the targeted, unit-specific interventions incorporated into the collaborative model were particularly effective in high-risk settings, significantly reducing the burden of MDRO infections in both ICU environments while preserving low incidence in general care areas.

**Table 3:** Comparison of MDRO infection incidence rates in ICU, ED-ICU, and general wards before and after implementation.

Unit	Pre-implementation	MDRO infections	Incidence rate (%)	Post-implementation	MDRO infections	Incidence rate (%)	χ^2^	*P*-value
ICU	1080	16	1.48	1198	5	0.42	7.041	.008
ED-ICU	153	5	3.27	377	1	0.27	8.767	.003
General ward	79,978	16	0.02	86,651	11	0.01	1.372	.241
Total	81,211	37	0.05	88,226	17	0.02	9.175	.003

χ^2^ = chi-square test, ED-ICU = emergency department intensive care unit, ICU = intensive care unit, MDRO = multidrug-resistant organism, n = number of surveyed isolates.

## 9. Discussion

This study demonstrates that implementation of a multidisciplinary collaborative management model significantly improved adherence to infection control protocols, reduced the detection rates of MDROs, and lowered the incidence of MDRO infections in critical care settings. Importantly, the observed gains in compliance – such as department-level training, early handover, environmental cleaning, and PPE use – can be explained by several underlying mechanisms. Regular multidisciplinary ward rounds created accountability across departments, while structured training programs reinforced frontline staff knowledge and skills. In addition, integration of information technology, such as automated alerts and standardized rounding records, enhanced communication and continuity, ensuring that corrective actions were rapidly implemented and sustained. These elements highlight how cross-disciplinary collaboration strengthened the overall infection prevention culture of the hospital.^[9,10]^

The reduction in MDRO detection, particularly for CRE and Acinetobacter baumannii, reflects the synergistic impact of targeted antimicrobial stewardship and enhanced microbiological surveillance. Stewardship interventions guided by antibiogram data reduced unnecessary broad-spectrum antibiotic use, while real-time laboratory reporting enabled earlier identification and isolation of colonized patients, thereby interrupting transmission pathways.^[11,12]^ The marked decrease in infection incidence in the ICU and ED-ICU further illustrates that unit-specific interventions—such as strict catheter bundle adherence and intensified monitoring – are especially effective in high-risk environments where invasive devices and patient acuity amplify MDRO spread.^[13,14]^

Our findings are consistent with previous studies demonstrating the benefits of multidisciplinary interventions. For example, Smith et al reported a 25% reduction in MRSA bloodstream infections after implementing an integrated infection control committee, while Jones and colleagues observed a 30% decline in CRE isolation following combined stewardship and environmental measures.^[15,16]^ By encompassing a broader spectrum of MDROs and hospital-wide settings, our study extends this evidence and underscores the importance of formalized governance structures and clearly defined team roles in achieving sustained improvements.^[17,18]^

Several limitations must be acknowledged. First, the retrospective single-center design may introduce unmeasured confounding and could overestimate the intervention effect, as concurrent quality improvement initiatives or shifts in patient mix might also have contributed to the observed changes. Second, the absence of a parallel control group limits our ability to fully exclude temporal trends unrelated to the intervention. Third, resistance mechanisms were assessed only by phenotypic susceptibility, without molecular epidemiology, which restricts our ability to distinguish clonal transmission from independent emergence of resistance. Finally, as the study was conducted in a tertiary center with relatively robust resources, the feasibility and impact of this model in primary hospitals or resource-limited settings may differ. Challenges such as insufficient infection control staffing, limited laboratory capacity, or lack of IT infrastructure could hinder implementation.

Future studies should therefore adopt prospective, multicenter designs that integrate molecular epidemiological monitoring to track transmission dynamics, conduct long-term follow-up to assess sustainability, and perform cost-benefit analyses to guide resource allocation. Such work would provide a stronger evidence base for scaling multidisciplinary collaborative models across diverse healthcare systems.

In conclusion, this study demonstrates that a structured multidisciplinary collaborative management model can improve compliance with infection control measures, reduce MDRO detection and infection rates, and achieve particularly pronounced benefits in high-risk wards such as ICUs. These findings provide practical evidence to support adoption of integrated infection control strategies in hospitals seeking to address the growing challenge of MDROs.

## 10. Conclusions

The multidisciplinary collaborative management model improved adherence to infection control practices and reduced both MDRO detection and infection rates, particularly in critical care units. This approach demonstrates a practical and effective strategy for mitigating HAIs caused by MDROs.

## Author contributions

**Conceptualization:** Lan Ding.

**Data curation:** Fubo Gao.

**Formal analysis:** Fubo Gao.

**Investigation:** Lan Ding.

**Methodology:** Lan Ding.

**Supervision:** Lan Ding.

**Validation:** Lan Ding.

**Visualization:** Lan Ding.

**Writing – original draft:** Lan Ding.

**Writing – review & editing:** Lan Ding.
